# A qualitative study into the difficulties experienced by healthcare decision makers when reading a Cochrane diagnostic test accuracy review

**DOI:** 10.1186/2046-4053-2-32

**Published:** 2013-05-16

**Authors:** Zhivko Zhelev, Ruth Garside, Christopher Hyde

**Affiliations:** 1 Peninsula Technology Assessment Group (PenTAG), University of Exeter Medical School, University of Exeter, Veysey Building, Salmon Pool Lane, Exeter EX2 4SG, UK; 2 European Centre for Environment and Human Health, University of Exeter Medical School, University of Exeter, Knowledge Spa, Royal Cornwall Hospital, Truro TR1 3HD, UK

**Keywords:** Cochrane reviews, Diagnostic accuracy, Sensitivity and specificity, Qualitative research, Think -aloud interview

## Abstract

**Background:**

Cochrane reviews are one of the best known and most trusted sources of evidence-based information in health care. While steps have been taken to make Cochrane intervention reviews accessible to a diverse readership, little is known about the accessibility of the newcomer to the Cochrane library: diagnostic test accuracy reviews (DTARs). The current qualitative study explored how healthcare decision makers, who varied in their knowledge and experience with test accuracy research and systematic reviews, read and made sense of DTARs.

**Methods:**

A purposive sample of clinicians, researchers and policy makers (n = 21) took part in a series of think-aloud interviews, using as interview material the first three DTARs published in the Cochrane library. Thematic qualitative analysis of the transcripts was carried out to identify patterns in participants’ ‘reading’ and interpretation of the reviews and the difficulties they encountered.

**Results:**

Participants unfamiliar with the design and methodology of DTARs found the reviews largely inaccessible and experienced a range of difficulties stemming mainly from the mismatch between background knowledge and level of explanation provided in the text. Experience with systematic reviews of interventions did not guarantee better understanding and, in some cases, led to confusion and misinterpretation. These difficulties were further exacerbated by poor layout and presentation, which affected even those with relatively good knowledge of DTARs and had a negative impact not only on their understanding of the reviews but also on their motivation to engage with the text. Comparison between the readings of the three reviews showed that more accessible presentation, such as presenting the results as natural frequencies, significantly increased participants’ understanding.

**Conclusions:**

The study demonstrates that authors and editors should pay more attention to the presentation as well as the content of Cochrane DTARs, especially if the reports are aimed at readers with various levels of background knowledge and experience. It also raises the question as to the anticipated target audience of the reports and suggests that different groups of healthcare decision-makers may require different modes of presentation.

## Background

Over the past 20 years, the Cochrane Collaboration has become one of the largest and most reputable sources of evidence-based healthcare information in the world. Its ambition to systematically identify, review and update the existing evidence in the field of healthcare research has been extended to making this information available and accessible to a diverse group of decision makers, such as healthcare practitioners, policy makers and consumers. While significant efforts have been made to improve the accessibility of the Cochrane systematic reviews of interventions (hereafter referred to as ‘Cochrane intervention reviews’), very little is known about the accessibility of the newcomer to the Cochrane library - the diagnostic test accuracy reviews (DTARs).

DTARs are systematic reviews of diagnostic test accuracy studies - primary studies that assess the ability of diagnostic tests to correctly identify a disease or other target condition by measuring the test’s error rate. In test accuracy studies, the results from the evaluated diagnostic test (index test) are compared with those from a reference standard - a diagnostic test or a combination of tests considered to be the most reliable way of diagnosing the target condition and assumed to be 100% accurate. The discrepancies in the results are used to measure the diagnostic accuracy of the index test as compared to the reference standard. The study may also have a comparative design, in which case two or more index tests are evaluated, hopefully against the same reference standard. A range of test accuracy statistics is derived from the resultant contingency table, in order to summarize and communicate different aspects of the test’s ability to identify the target condition (Table 1). The measures most commonly reported in the literature are the test’s sensitivity and specificity, which represent the proportions of correctly diagnosed diseased and non-diseased patients, respectively. However, in some cases other test accuracy measures, such as diagnostic odds ratios, predictive values and likelihood ratios, may be more relevant, depending on the type of decisions the information will be used for [1].

**Table 1:** Two-by-two table and derivative test accuracy measures

		**Reference standard (disease status)**		**Test accuracy measures ↓**
		**Disease present**	**Disease absent**	
Index test (test under evaluation)	Positive result	True positive (TP)	False positive (FP)	Positive predictive value = TP/(TP+FP)
	Negative result	False negative (FN)	True negative (TN)	Negative predictive value= TN/(TN+FN)
Test accuracy measures →		Sensitivity = TP/(TP+FN)	Specificity = TN/(TN+FP)	Diagnostic odds ratio = (TPxTN)/(FPxFN)

Test accuracy information is used by policy makers, practitioners and researchers in a wide range of decision-making contexts such as:

• Making decisions about the introduction of new diagnostic tests;

• Evaluation of the effectiveness and efficiency of current diagnostic practices;

• Development of more effective and efficient diagnostic strategies, and

• A range of clinical decisions such as whether the test should be ordered and the probability of the target condition in individual patients giving a positive or a negative test result.

Like all other research, test accuracy evaluations are prone to bias and their use in decision making requires, as a preliminary step, a rigorous assessment of their validity and applicability. It has been demonstrated that such studies often suffer from serious methodological shortcomings; their sample sizes are usually too small to allow the calculation of precise estimates of test accuracy; and they are poorly reported, which makes the assessment of their methodological quality and applicability difficult [2-7]. Also, a number of studies have shown that both healthcare professionals and consumers find test accuracy information confusing and struggle to apply it correctly when interpreting the results from diagnostic tests [8-26].

The application of systematic review methods to test accuracy evaluations allows for a more comprehensive, rigorous and transparent way of gathering, appraising and analyzing the existing evidence and, in some cases, enables the calculation of more accurate estimates by pooling the results from several individual studies [27]. Although such methods hold the promise of producing better quality information, they have their own limitations, biases and complexities and conveying their results to a diverse audience is equally challenging.

Over the past few years, the Cochrane Collaboration has been developing its methods for systematic reviews of test accuracy studies, with the first review being published in 2008 and an increasing number of DTARs being published since then. As for all Cochrane reviews, the expectation within the Cochrane collaboration is that the Cochrane DTARs should be accessible to a wide range of healthcare decision makers across the world. At present, however, very little is known about the difficulties that different stakeholders in the healthcare system might experience when reading and trying to make sense of a Cochrane DTAR and, to our knowledge, no research has yet been done in the accessibility of this type of review.

To start filling this gap, we conducted a qualitative study investigating UK-based healthcare decision makers’ experience of reading, and making sense of, a Cochrane DTAR. The current paper reports on the main findings and discusses possible ways of making Cochrane DTARs more accessible. It also raises the question about the target readership of these documents and the need to use different formats to convey the results from the Cochrane DTARs. Although the focus of the study is specifically on the accessibility of the Cochrane reviews, many of the findings are relevant to and could be applied to improve the accessibility of DTAR reports in general, and to other types of systematic reviews.

This project was funded by a NIHR Cochrane - NHS Engagement Award. Visit the NETSCC website for more details (http://www.netscc.ac.uk/). The views and opinions expressed therein are those of the authors and do not necessarily reflect those of the Department of Health. The study received an ethical approval from the local NHS research ethics committee.

## Methods

In order to explore the processes through which healthcare professionals make sense of a DTAR and the difficulties, if any, they encounter with different aspects of the review, we conducted a series of interviews based on the think-aloud interviewing technique. The think- aloud interview is a method widely used in psychological and social research when an understanding of the cognitive processes involved in the completion of a specific task, such as reading and making sense of a text, is sought [28-31]. It consists of asking participants to verbalize their thoughts while working on the task, thus making ‘visible’ the cognitive aspects of the problem-solving process. The interview is recorded and transcribed verbatim and, depending on the objectives of the study, a variety of methods for analysis have been proposed [28-35]. Although, this technique has gained significant popularity in the study of text comprehension, its application in studying the understanding of intact, naturally- occurring texts, especially complex and voluminous texts such as systematic reviews, has been limited [28]. For the purposes of the current study we adapted the method so that we could capture not only participants’ verbalizations but also their non-verbal interaction with the text. We assumed that participants’ understanding of the reviews would depend on a range of factors that could be grouped into the following three categories: background knowledge and experience, characteristics of the text and characteristics of the task. Keeping the task constant, we explored the interaction between background knowledge and text presentation using three different reviews and interviewing a purposive sample of healthcare decision-makers with diverse background knowledge.

As interview material, we used the following Cochrane DTARs:

• ‘Galactomannan detection for invasive aspergillosis in immunocompromized patients’ [36].

• ‘Magnetic resonance imaging versus computed tomography for detection of acute vascular lesions in patients presenting with stroke symptoms’ [37].

• ‘Physical examination for lumbar radiculopathy due to disc herniation in patients with low back pain’ [38].

For shortness, we will refer to them here as the Galactomannan review, the MRI versus CT review and the Physical examination review, respectively. We chose these particular reviews because at the time of conducting the study they were the only full text DTARs published in the Cochrane library. Further details of the reviews are provided in Table 2.

**Table 2:** Details of the three DTARs used as interview material

	**Galactomannan review**	**MRI versus CT review**	**Physical examination review**
**Index test(s)**	Galactomannan sandwich ELISA (Platelia©)	Diffusion-weighted MRI and CT (for the diagnosis of acute ischemic stroke); MRI (for the diagnosis of acute hemorrhagic stroke)	A number of physical examination tests such as straight leg raising and crossed straight leg raising tests.
**Reference standard**	EORTC/MSG criteria (see review)	Acute ischemic stroke: a combination of clinical and imaging information supported by clinical or imaging follow-up (CT or MRI) or autopsy;	Diagnostic imaging or findings at surgery
		Acute hemorrhagic stroke: a clinical diagnosis supported by CT or autopsy	
**Target condition**	Invasive aspergillosis	Acute ischemic stroke; acute hemorrhagic stroke	Radiculopathy due to lower lumbar disc herniation
**Presentation of the results from meta-analysis**	Estimates of sensitivity and specificity (percentages) and false positive and false negative rates (natural frequencies)	Estimates of sensitivity and specificity (percentages)	Estimates of sensitivity and specificity (percentages)

### Participants and sampling

Participants’ selection was based on the results from an online survey reported here as an additional file [see Additional file 1]. We used the survey to gather preliminary information about prospective participants’ familiarity with diagnostic test accuracy (DTA) concepts and methods, systematic review methodology and the three DTARs chosen as interview material. Prospective participants in the survey were clinicians to whom the subject areas of the reviews were of relevance, and policy makers and researchers involved in decision making about diagnostic tests. We decided to exclude consumers and carers, not because we believe that this type of review is not relevant to them, but because at present the Cochrane DTARs contain no Plain language summary, which already suggests that in their current format the reviews are not intended for non-specialist audience. We acknowledge, however, that diagnostic accuracy is relevant to everybody who makes decisions about diagnostic tests, including consumers and carers, and that, ultimately, the results of the Cochrane DTARs should be made accessible to all of them, even though this may require different formats and presentations.

Prospective participants were contacted via professional organizations and networks and, after completing the survey, were asked whether they would like to take part in an interview. To ensure we understood a range of perspectives, we purposively sampled people with high or low levels of experience in four key spheres: the clinical area, DTARs, systematic review methods generally and policy making. The volunteers selected for interviewing were allocated to one of the three reviews, with the clinicians being allocated to the reviews relevant to their specialty and the participants with non-clinical backgrounds allocated so as to balance the three groups in terms of participants’ clinical and methodological knowledge (Table 3).

**Table 3:** Participants background and experience

**Review**	**Professional background (code used in the quotes)**	**Knowledge of the clinical area**	**DTA-related knowledge**	**Systematic review methods**	**Academic role within university**	**Policy- making experience**
Galactomannan detection for invasive aspergillosis in immune-compromized patients	Hematologist (H1)	Yes	Good	Some	Yes	Yes
	Hematologist (H2)	Yes	Good	Some	Yes	Yes
	Policy maker (PM1)	No	Some	Good	Yes	Yes
	Systematic reviewer (SR1)	No	Little	Good	Yes	No
	Health economist (HE1)	No	Little	Good	No	No
	Systematic reviewer (SR2)	No	Good	Good	Yes	Yes
Physical examination for lumbar radiculopathy due to disc herniation in patients with low back pain	GP (GP1)	Yes	Little	Little	No	No
	GP (GP2)	Yes	Little	Little	No	No
	Physiotherapist (PH1)	Yes	Little	Good	Yes	No
	GP (GP3)	Yes	Good	Good	No	Yes
	Physiotherapist (PH2)	Yes	Good	Good	Yes	Yes
	Policy maker (PM2)	Yes	Good	Good	Yes	Yes
	Policy maker (PM3)	No	Some	Good	Yes	Yes
MRI versus CT for detection of acute vascular lesions in patients presenting with stroke symptoms	Radiologist (R1)	Yes	Little	Little	No	No
	Systematic reviewer (SR3)	No	Little	Some	No	No
	Health economist (HE2)	No	Good	Good	No	Yes
	Health economist (HE3)	No	Little	Some	No	No
	Systematic reviewer (SR4)	No	Little	Good	Yes	No
	Systematic reviewer (SR5)	No	Good	Good	Yes	No
	Systematic reviewer (SR6)	Yes	Some	Good	Yes	No
	Policy maker (PM4)	Yes	Good	Good	Yes	Yes

Legend: DTA-related knowledge and systematic review methods: ‘Little’ - the participant has poor understanding of basic DTA concepts such as sensitivity and specificity/little understanding of systematic review methods; ‘Some’ - good understanding of basic DTA concepts/general idea of systematic review methods; ‘Good’ - familiar with DTA theory/systematic review methods.

### Interview procedure

At the beginning of the interview, participants were helped to practice the think-aloud technique on a sample of informational text [28-30]. The instructions for the main task were as follows. Participants were asked to imagine that they were members of a policy making committee which was considering the application of the evaluated tests. They had been asked to read the selected review and to feed back to the committee the points they considered most important, with the supporting evidence. This scenario was chosen because it was relevant to a wide range of healthcare professionals including policy makers, researchers and clinicians. We did not ask clinicians to apply the review results to a clinical scenario as this would have required additional information, such as information concerning patient outcomes, which was not available in the reviews. Participants were instructed to think aloud while reading the review specifically, to tell the researcher what they were getting from the text, what they were doing and any other thoughts that came to mind. We did not place any restrictions on the participants’ verbalizations and encouraged them to verbalize everything that came to mind while working on the task [34]. We made it clear to them, however, that we were interested in their thoughts emerging *in relation* to the task they were performing rather than in general comments or explanations. Participants were also told that there was no set time within which they had to complete the think-aloud task. They were asked, however, to allow an extra 15 minutes for additional questions and for closing the interview. The interviews were video-recorded with the digital camera set in such a way as to capture the page(s) being referred to and the participants’ actions with the text, such as leafing through the pages, highlighting, pointing to specific passages and so on. The video recording allowed easier synchronization of the verbal protocol with the parts of the document being used and better documentation of the non-verbal behaviour, which would have been difficult if only audio recording was used.

Participants received a full single-sided color-printed copy of the review they were allocated and a pen, highlighters and paper. They were told that if they wished, they could take notes or write directly on the document. At the end of the interview, the review and the participants’ notes were collected to be used in the analysis.

Once the think-aloud part of the interview was completed, participants were asked to interpret specific elements of the review such as the Methodological Quality Graphs, the forest plot and/or the SROC plot, if they had not done so during the interview. Participants were also asked a number of questions clarifying:

• Their experience with diagnostic test accuracy information, especially in terms of using such information in their everyday practice;

• Their experience with DTARs and other types of systematic reviews; and,

• Their knowledge of the subject area including experience with the evaluated tests.

Interview records were transcribed verbatim and segmented into sections that followed the structure of the review, such as ‘abstract’, ‘background’ and so on. The segmented transcripts, together with the video records, were imported into NVivo (a qualitative research software; NVivo v.8, QSR International) and synchronized. Each interview was watched section by section and a description of any significant non-verbal behaviour was added to the transcript. Inductive coding - coding based on the themes emerging from the data [39] - was carried out, focusing on three broad categories of events:

• Participants’ interaction with the text, such as the sequence and manner of reading, read/ignored text or diagrams, non-verbal expressions of hesitation, confusion and so on;

• Interpretation of information; and,

• Errors, difficulties and problems.

### Analysis

The interviews were then analyzed by comparing participants’ behavior, interpretations and the difficulties they experienced at each section of the document, within and across the three reviews, and between different categories of participants. The whole process was highly iterative, involving repeated watching of the interviews, or segments of them, and going back and forth between different stages. In order to ensure that participants’ verbalizations were interpreted correctly, two researchers watched and analyzed a sample of the interviews. Specific examples, especially those related to errors and difficulties, were discussed at regular team meetings and with members of the steering group, who are specialists in DTAR methodology.

## Results

We estimated that the invitation was sent to more than 1,200 healthcare professionals, of whom only 103 completed the survey (< 10% response rate) and only 46 volunteered to take part in an interview (< 5%), with some of them subsequently pulling out due to busy schedules or other reasons. The volunteers tended to have greater knowledge of test accuracy and systematic review methods and to be involved in teaching or research (see Additional file 1). We selected interview participants aiming to achieve maximum variability in terms of background knowledge and experience with the subject area, systematic review methods, DTA concepts and methods and use of research evidence in policy making. We stopped conducting more interviews when new themes stopped emerging and sufficient data was available on the ones already identified. Eight of the included participants were clinicians and 13 had non-clinical background. In terms of specialty, we interviewed one radiologist, two hematologists, three general practitioners and two physiotherapists. Of the non-clinicians, six were systematic reviewers, three were health economists and four were members of local and/or national policy making bodies (see Table 3). The interviews took place in venues chosen by the participants, usually their offices, and took between 30 minutes and one hour to complete the ‘think aloud’ task with the whole interview taking between one and one and a half hours.

Below we present the interview results by looking at how different categories of participants read the reports, the range of difficulties they experienced and the impact that difficulties, reading strategies and presentation of information in the reports had on their overall understanding of the reviews. Individual participants are identified throughout the text by unique codes that can be linked back to their background characteristics presented in Table 3.

### Reading patterns

The abstract was used as a ‘gateway’ to the review - participants read it first in order to ascertain the relevance of the review and its potential to inform clinical and policy decisions. The reading of the reviews was highly selective, with the employed reading strategies reflecting participants’ perceptions of accessibility, relevance and importance. Differences were observed between those who were familiar with the methodology of systematic reviews (PM1, SR1, HE1, SR2, PH1, GP3, PH2, PM2, PM3, HE2, SR4, SR5, SR6, PM4) and those who had little experience with this type of research (GP1, GP2, R1). The former group of participants knew broadly what to expect in the report, they were more strategic when choosing what and how to read and their reading was more iterative, resembling the process of putting together a jigsaw puzzle: they prioritized their reading and looked for information they considered relevant and important, such as inclusion and exclusion criteria, reference standard and methodological quality assessment; and tended to ignore those aspects of the review they knew were standard for all Cochrane reviews, such as data extraction and management procedures. They tried to make sense of the review by piecing together different bits of information and identifying missing elements, and comparing the emerging picture with their understanding of a well-conducted systematic review. In some cases, a lack of familiarity with methods specific to DTAR hampered participants’ understanding and led to misinterpretations and wrong conclusions, which will be discussed in the next section (PM1, SR1, HE1, PH1, SR4, SR6).

Participants who had limited understanding of systematic review methods - all of whom were non-academic clinicians (GP1, GP2, R1) in our sample - adopted a different strategy. After reading the abstract they stated that, in the real world, they would not read the rest of the review, since they were interested mainly in the results, considered the methodological aspects irrelevant or difficult to understand, and had limited time to read such papers. They read the reviews in a linear manner, starting from the beginning and reading through to the end, skimming over and skipping parts of the text they considered inaccessible or irrelevant for them as clinicians. They focused on sections that provided an accessible narrative summary of the results, such as the abstract and the conclusions section and consistently skipped the methods and results sections, which they found particularly difficult and/or considered irrelevant to the clinician’s role.

Participants’ understanding of the abstract strongly influenced their decisions on whether and how to read the rest of the review; and had an impact on their further interpretations of the text. In some cases, misunderstanding of information provided in the abstract remained unchecked due to participants skipping sections of the review and often resulted in further confusion and misinterpretations. This is considered in more detail below.

### Difficulties experienced when reading the DTAR

We grouped the difficulties experienced by participants in the following categories, those related to:

• The design of DTARs;

• The interpretation of results when presented as sensitivity and specificity;

• The review methods specific to DTARs; and,

• Poor layout and presentation of information in the reports.

#### Difficulties related to the design of DTARs

Half of the participants in the study had limited experience with diagnostic research and, as a result, poor understanding of its design. On the other hand, practically all participants had some experience with intervention reviews and, drawing on this knowledge, made assumptions about the design of DTARs which, in some cases, were wrong, and led to misunderstanding and confusion.

The most common error was to misinterpret the role of the reference standard as if it was a comparator. A number of participants (GP1, GP2, R1, HE3) acknowledged that they did not know the meaning of the terms ‘index test’ and ‘reference standard’ as used in the context of diagnostic accuracy research. As a result, they mistakenly assumed that the index test was compared to the reference standard in order to establish which of the two technologies was performing better. A related source of confusion was the wrong assumption that DTARs are comparative in nature, probably as a result of participants’ greater experience with comparative intervention reviews. Two factors in the presentation of the reviews contributed to the above problems. First, no glossary of key terminology was provided in the text and even when participants felt uncertain about the meaning of specific terms, they were unable to find a definition. Second, when the roles of the tests were not clearly and explicitly defined in the abstract, participants made assumptions, which were sometimes wrong and affected their understanding of the subsequent sections. Wrong interpretations often remained unchecked as a result of the participants skipping the methods and results sections of the review - and led to a misunderstanding of the final results.

For example, in the Physical examination review the diagnostic accuracy of different physical tests and their combinations was assessed for the diagnosis of disc herniation. The conclusions, as stated in the abstract, read: ‘When used in isolation, current evidence indicates poor diagnostic performance of most physical tests used to identify lumbar disc herniation. However, most findings arise from surgical populations and may not apply to primary care or non-selected populations. Better performance may be obtained when tests are combined’. (van der Windt *et al*. 2010, p2).

The roles of the different tests, however, were not explicitly defined in the abstract. One of the participants who read this review (PH1) - a clinician with some experience with intervention reviews but none with DTARs - after reading the abstract, wrongly assumed that the statement ‘Better performance may be obtained when tests are combined’ (van der Windt *et al*. 2010, p2) referred to a combination of physical tests (index test) and imaging (reference standard):

‘So what this is saying to me overall is that the sort of physical tests that you would use aren’t particularly good at actually diagnosing this disc herniation, but when they’re using imaging … err … tests in combination with it, the tests are slightly more accurate, but still not great.’ (PH1)

Since she skipped most of the methods and results sections, she remained under this impression until the very end of the reading and it was reflected in her final message. In this case, the lack of experience with diagnostic research, and the fact that the roles of the tests were not explicitly defined in the abstract, interacted with the participant’s reading strategy to result in a complete misunderstanding of the review.

This problem was encountered more frequently in participants reading the MRI versus CT review, which combined two different evaluations - comparative and non-comparative - with the computed tomography (CT) being an index test in the first evaluation and part of the reference standard in the second. This complexity, however, was not made prominent enough in the abstract and other sections of the review - in terms of wording and layout - and even participants with good knowledge of DTAR methods got the roles of the tests wrong at first and only after reading the whole review understood what was compared and why. One such participant, who initially misunderstood the design of the review, after reading the full text, commented:

‘The abstract was fairly vague. When reading the abstract, I thought they were comparing MRI - well, MRI with CT for acute stroke. I suppose when you are looking back at it, it makes more sense … but not straight away … I wasn’t clear what they were doing. It wasn’t clear what the reference standard was. It looked like the CT was the reference standard rather than imaging with clinical follow-up or autopsy.’ (SR5)

Participants less familiar with DTAR methods found this lack of clarity even more confusing and some of them failed to understand the design of the review even after reading the full text (R1, SR3, HE3).

#### Difficulties interpreting the results when presented as sensitivity and specificity

Participants unfamiliar with DTA research struggled to make sense of the review results when presented as sensitivity and specificity and experienced the following difficulties:

‘And then what I am also learning is that OK, it is quite specific, and I always get specificity and sensitivity mixed up, probably because I don’t teach it, and I always have to go back and look it up, but I think ‘specific’ means that if it is there, it will demonstrate it but it is also not particularly sensitive and has a high false negative. I don’t know if that is right or not, but there we are.’ (GP1)

• They could not recall the definitions for sensitivity and specificity and spontaneously commented that, since they did not come across these terms very often, they always found them confusing and every time had to look them up:

Limited use of test accuracy information in clinical practice was reported by most of the clinicians who took part in the study, which seems to explain the observation that non-academic clinicians (R1, GP1, GP2) found DTA information more difficult to interpret when compared to their colleagues involved in teaching (H1, H2, PH2).

‘So more sensitive … more sensitive tests would mean that … would find out more positive … if they’re … yeah … I’m rubbish at this, so I have to write a little table (draws 2×2 table). So if you’ve got a true positive, if you’ve got lots of true positives, even if you get a few false positives that’s sensitivity. And if you have a high specificity means that the positives you do have are really all proper positives and you haven’t got many … many … mmm … many … false … aaah, stuck now. Aaah, I don’t know (laughing nervously).’ (R1)

• Participants confused sensitivity (the proportion of test-positive among those with the disease) and specificity (the proportion of test-negative among those without the disease) with the positive and negative predictive values (the proportion of diseased among those with a positive test result and non-diseased among those with negative test result, respectively).

In the above excerpt, the participant wrongly assumed that sensitivity refers to the proportion of true positives among those with positive test results (which is the positive predictive value), while in fact it refers to the proportion of true positives among those who have the disease.

‘Now I am racking my brain to try and remember how to interpret these (forest plots), which I should be able to, and my brain says that if it crosses 1 … or 0 … it is not relevant. I can’t remember. I will wait till I am told. So there is a lot of heterogeneity (reading the text) so … OK, I am slightly confused at this stage!’ (GP2)

• Participants misinterpreted sensitivity and specificity estimates by wrongly applying heuristics that they had learnt in relation to ratio measures. More specifically, when interpreting the confidence intervals of sensitivity and specificity, participants looked to see whether or not the confidence interval was crossing one. In the context of ratio measures, if the confidence interval crosses one this would mean that the difference between the compared interventions could be due to a chance and, therefore, is not statistically significant. Participants mistakenly transferred this rule when interpreting the confidence intervals of sensitivity and specificity and wrongly concluded that a confidence interval reaching 1.00 (100% sensitivity or specificity) indicated poor test performance. This type of misinterpretation was encountered both when participants interpreted the summary estimates and when interpreted the forest plots, as illustrated in the following excerpt:

This type of error was encountered only in non-academic clinicians who had little experience with both intervention reviews and DTARs (GP1, GP2, R1).

• Some participants tried to infer as to whether the evaluated tests would be useful as rule-in or rule-out tests (H1, H2, SR2, GP1, GP3, PH2, PM2, R1, HE2, SR5, PM4). In principle, a rule-in test needs to be very specific so that the rate of false positives is low and a positive test result is more likely to be a true positive; a rule-out test, on the other hand, needs to be very sensitive so that the rate of false negatives is low and a negative test result is more likely to be a true negative. Since this is counterintuitive, some participants (R1, GP1, GP3) got confused and wrongly dismissed tests that had lower sensitivity as useless for ruling in the condition, even when they were very specific and, therefore, perfect for this role. Such mistake was made even by a participant (GP3) who had in the past been involved in diagnostic accuracy research. In this case, the misinterpretation was due to ‘rusty’ knowledge rather than to a lack of understanding. It shows, however, that such mistakes are easy to make, especially when the knowledge is not routinely used in everyday practice.

• Another, more general issue, reflecting participants’ statistical literacy rather than their knowledge of DTAR methods, was their tendency to ignore the confidence intervals when interpreting the summary estimates for sensitivity and specificity. By doing so, some participants (R1, SR3, HE3) came to the wrong conclusion that one of the compared tests was better than the other while, in fact, the wide and overlapping confidence intervals suggested that there was a significant level of uncertainty and no definite conclusions could be drawn from the results. This problem was exacerbated when the statistical uncertainty was not explicitly discussed in the authors’ interpretations of the results.

Presenting the results as positive and negative error rates expressed as natural frequencies (Galactomannan review) greatly improved participants’ understanding:

‘Err, the overall sensitivity 78%, specificity 81%. Err … okay, so sensitivity picks up how many … err…I always get muddled up between the difference between sensitivity and specificity (laughs). Okay … Okay, conclusions - a cut-off value 0.5. Yeah. That’s what the consequences would be. You would have … two patients missed if you had 8% prevalence, 17 patients will be treated unnecessarily. Okay. If you use a cut-off of 1.5, you would miss three, treat wrongly five. Okay. But the results were very heterogeneous.’ (HE1)

The above quote shows that even participants with limited understanding and experience with test accuracy, who initially struggled to make sense of the information when presented as sensitivity and specificity, immediately grasped the implications of the results when the same information was presented as natural frequencies. Such presentation was found useful even by participants with relatively good understanding of DTAR methods, as illustrated by the following excerpt:

‘And they have actually concluded that if you have a low level, 0.5 (cut off), a disease prevalence of 8%, two patients will be missed, that’s 2% presumably, of 100 patients. Seventeen patients will be treated unnecessarily. If they go up to 1.5 (cut off), that will mean three patients will be missed and five will receive unnecessary treatment. And that’s actually quite a useful way of actually presenting the data relating to sensitivity and false positives, false negatives.’ (H1)

This form of presentation was welcomed by everybody who read the Galactomannan review and was suggested as a good way of communicating the results to a non-expert audience.

#### Difficulties related to a lack of familiarity with review methods specific to DTARs

Participants who had limited experience with DTARs and little knowledge of the methods specific to this type of review experienced a number of difficulties when reading the methods section and trying to make sense of the results.

##### Study design

Participants who had limited experience with diagnostic accuracy research (those with ‘little’ or ‘some’ DTA-related knowledge in Table 2) were not familiar with different types of study design used in diagnostic research and did not know what the implications of including or excluding specific designs were:

‘What I’m thinking now is ‘Are they the sort of studies that I would think would be best for a diagnostic test? I don’t look at diagnostic tests often, so I don’t know. I’m used to looking at treatments, so I’d normally want to see randomized controlled trials. Case control study? Sounds OK. Cross-sectional studies. But I’d probably want to do a little extra reading around; is this the type of thing that’s usually looked at for diagnostic tests?’ (PM1)

##### Methodological quality assessment

With a few exceptions (SR2, PM2, HE2, SR5), most of the participants were unfamiliar with the Quality Assessment tool for Diagnostic Accuracy Studies (QUADAS) and, as a result, struggled to make sense of the assessment [40-42]. The definitions of the individual assessment criteria provided in the reviews focused on the way in which the QUADAS items had been customized for the purpose of the particular review and assumed prior knowledge of the original QUADAS criteria. Participants unfamiliar with QUADAS found these definitions largely inaccessible and tended to skip them:

(Participant): ‘I’m just looking at these headings (of the methodological criteria), I’ve got no idea what they’re going to include under those paragraphs headings (browsing over the text without reading through).’

(Researcher): ‘Can you pick one and try to see when you read it through whether it makes sense?’

(Participant): ‘Okay, let’s go for partial verification prevented (reading) … No, that’s saying nothing to me at all. Let’s try differential verification avoided (reading)… No …’

(Researcher): ‘So does it get clear when you read it (the definition)?’

(Participant): ‘No … It’s not … err … it’s not explained to me clearly. I guess because it’s a … specialist thing - you’ve got a specialist condition, specialist test, and diagnostics is not something that we routinely have to think about, and therefore I really need something written aimed at a much, much lower level, because this is just too technical.’ (PM1 reading the Galactomannan review)

Having failed to make sense of the provided definitions, participants tended to ignore the unfamiliar methodological criteria, such as those related to verification and incorporation biases, and instead focused on items they were familiar with, such as blinding. The definitions were presented differently in the three reviews. In the Galactomannan review a list of detailed definitions was provided within the methods section; in the MRI versus CT review only brief explanations of some of the criteria were given in the text, without visually separating them from the rest of the paragraph; and in the Physical examination review the list of criteria was given in an appendix. Most successful in ‘attracting’ participants’ attention was the list of criteria provided in the Galactomannan review and least successful was the appendix in the Physical examination review.

Participants’ approach to the methodological quality assessment depended on their experience with systematic reviews, as well as on their knowledge of the specific QUADAS criteria. Those with little experience tended to skip the section or to make very general comments often driven by the color coding of the methodological quality diagram. For instance, they made comments such as ‘lots of red’ or ‘lots of green’, without understanding what the implications of not meeting a particular criterion were. The ‘traffic light’ color coding was sometimes interpreted subjectively, with participants focusing on the ‘red’ (poor quality) and ‘green’ (good quality) and ignoring the items coded ‘yellow’:

‘Basically what I would do if I was going to look at it, I would look for the red dots and see what they missed, and see how many greens there were.’ (PH2)

Also, the lack of key to the diagram in the Physical examination review led to some participants interpreting the ‘yellow’ code as ‘not too bad’ rather than ‘unclear’ (its correct meaning) and combining the items coded ‘yellow’ and ‘green’ into ‘better quality studies’ category. Other presentation issues also impacted on participants’ understanding of this section of the review. For instance, all participants focused on the diagram and skipped the associated text, which was placed on the following page. They considered the text redundant while, in fact, it contained important details about the performance on specific criteria, which the participants missed.

##### Statistical analysis and data synthesis

Only participants with expert levels of knowledge were familiar with the statistical methods employed in the review, and only they read in full the ‘Statistical analysis and data synthesis’ section. The rest of the participants either completely skipped or skimmed over this section expressing familiarity with the 2×2 table, the forest plots and, in some cases, with the ROC plot and stating that they lacked the necessary expertise to discuss the appropriateness of the statistical methods.

When reading the findings section of the review, participants who had poor understanding of basic DTA concepts such as sensitivity and specificity struggled to interpret the forest plots or misinterpreted the presented information (GP1, GP2, SR3, SR4, HE3). For instance, participants who misread the confidence intervals of sensitivity and specificity estimates as if they were estimates of ratio measures, made the same mistake when interpreting the forest plots and either got confused and gave up, or misinterpreted the information (R1, GP1, GP2, SR3, SR4).

**Figure 1:**
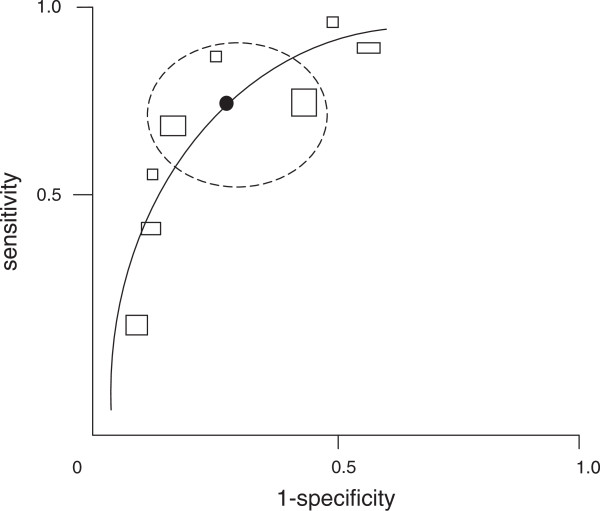
**An example of a Summary Receiver Operating Characteristics (SROC) plot of sensitivity versus 1-specificity.** Each rectangle represents the results from a single study; the width and the height of the rectangles could be used to represent the number of patients with and without the target condition; the solid line is the summary ROC curve; the thick black spot is the mean value for sensitivity and specificity; the ellipse around the black spot represents the 95% confidence intervals around the summary estimate.

Participants with limited DTA knowledge skipped the SROC plot (Figure 1) either because they were unfamiliar with this type of diagram or because they were convinced that it was extremely difficult to interpret. A lack of detailed legend contributed to this decision and significantly hindered the interpretation.

‘I mean, from that, there’s no key to tell me what all these different boxes mean on this particular diagram. So it doesn’t … I presume these are different studies, but there’s nothing to tell me what anything means on this … on this particular diagram. Err … so if I was to guess, these are different … individual studies looking at the sensitivity and specificity, and the size of box, I guess, is to do with the sample size. I don’t know what the black dot means, or this circle in the middle, the highlighted bit. So for me, I would … because I’ve never seen this diagram before, what I would do is look at the text in terms of this. But whether this tells me any more (turning page) than the previous forest plots, I don’t know.’ (PH1)

Even participants who had come across an ROC plot before found the SROC difficult to interpret and skipped it when no key was available (GP3, PM3). When a detailed legend was provided and the associated text contained accessible interpretation even participants unfamiliar with the SROC plot managed to interpret it reasonably well (PM1, SR1, HE1, GP1).

#### Difficulties related to layout and presentation

Layout and presentation played an important role in enabling or hindering participants’ understanding of the reviews. The most important presentation weaknesses that had direct impact on participants’ understanding were as follows:

• Figures had no key, which made them difficult to interpret;

• Important information was not made prominent enough (through highlighting or bulleting) and, as a result, was easy to omit;

• There was a mismatch in the data provided in different parts of the review, such as disagreement in the values provided in the abstract, the results section and the summary of results table, which led to confusion especially in participants who were less confident in their understanding of the review;

• The reviews contained no glossary and participants could not check the definitions of unfamiliar terms.

Other issues had less direct impact on participants’ understanding but affected their motivation to engage with the text and their choices about what and how to read. In general, poorly-presented and difficult to understand information was often skipped and, in some cases, the following sections were also skipped as a result of this. Here are some of the identified problems:

• Figures and tables were cluttered and difficult to read;

• Tables had no orphan control and were difficult to follow;

• Figures and associated text were disconnected and, in some cases, the text did not relate to the figure on the same page;

• The table of contents was not detailed enough to guide participants unfamiliar with the structure of review reports.

### Final messages and overall understanding

After reading the review, participants were asked to report - as if to a policy-making committee - the points they considered most important. Those of them who had little experience with systematic reviews and poor understanding of test accuracy concepts, found the reviews largely inaccessible, skipped large parts of the report and based their final messages almost entirely on the authors’ conclusions (GP1, GP2, R1, SR3, HE3, SR4). Their messages often contained errors, such as misunderstanding the roles of the tests; misinterpreting sensitivity and specificity estimates and their implications for practice; ignoring the statistical uncertainty in the results and poor understanding of the limitations of the review. Within this category of participants, clinicians with little knowledge of research methods (GP1, GP2, R1) tended to ignore the caveats and complexities and ‘translate’ the results into less ambiguous statements from which clear implications for practice could be formulated. Also, the boundary between their own preconceptions and experiences with the topic and the results from the review was often blurred, leading to conclusions unsupported by the review results.

For instance, the conclusions section in the abstract of the Physical examination review read:

‘When used in isolation, current evidence indicates poor diagnostic performance of most physical tests used to identify lumbar disc herniation. However, most findings arise from surgical populations and may not apply to primary care or non-selected populations. Better performance may be obtained when tests are combined’. (van der Windt *et al*. 2010, p2)

One of the clinicians reading this review concluded in his final message the following:

‘And the conclusions it comes to I would entirely agree with, which is that it looks likely that the combination of different techniques in examination, whether that be straight leg raising or crossed straight leg raising or whatever, would result in better diagnostic accuracy and hence a more accurate referral. So the patient gets a better service if we are teaching people, or ensuring that people do a combination of examination techniques.’ (GP1)

This participant skipped most of the methods, results and discussion sections, either because he considered them irrelevant to him as a clinician or because he found them difficult to understand. As a result, he missed the fact that there was not much evidence to support the statement that better performance could be achieved by combining different tests and no clarity as to which tests should be combined and how to aggregate their results. Although this was reflected in the conditional form of the sentence in the authors’ conclusions (see quotation above), he not only ignored the uncertainty but also jumped to the conclusion that combining tests will lead to better practice, a claim made nowhere in the review.

Participants familiar with systematic review methods tried to conduct a critical appraisal of the review and to compare their own conclusions with those of the review authors. Their capacity to do so depended, however, on their knowledge of and experience with DTARs. For some of them (PM1, SR1, HE1, PH1, SR4, SR6) specific aspects of the review, such as the methodological quality of the included studies and the SROC plot, remained difficult to understand. As a result they tended to focus on familiar aspects and their final messages contained some of the errors discussed above. On the other hand, participants familiar with the methodology of DTAR (H1, H2, SR2, GP3, PH2, PM2, HE2, SR5, PM4) compared, in their final messages, their own conclusions with those of the review authors and, in some cases, pointed out that they would not have come to the same conclusions, based on the data presented in the report. Some of them (SR5, SR6, PM4) found the conclusions in the abstract to be more cautious and consistent with the review results than the conclusions in the main body of the review which, in their view, contained statements not supported by the presented evidence (MRI versus CT review). The clinicians in this group (H1, PH2, GP3) -who read the reviews very carefully - also discussed the results with regards to their implications for clinical practice. They were, however, more cautious in their messages and emphasised the limitations of the results both in terms of their internal and external validity.

Only those participants who read the Galactomannan review produced messages in which the accuracy of the evaluated tests was discussed more specifically and in numerical terms:

‘Err … so I didn’t get sucked into not being able to understand those ROC curves, I’d probably be looking for some textual information around there and I would be … quoting verbatim … err … where people have said at (cut off) 0.5 you miss out two (patients) but treat 17 extra, if you use (cut off) 1.5 you miss out three (patients) but only treat five unnecessarily.’ (PM1)

As the above excerpt shows, this presentation format allowed participants not only to understand what exactly specific test accuracy results meant but also to communicate this information to other people, a difficulty often mentioned by the participants.

## Discussion

The application of the Cochrane review methodology to test accuracy studies allows for a comprehensive, systematic and rigorous evaluation of the existing evidence and, in cases where the pooling of individual studies is appropriate, enables the calculation of more precise test accuracy estimates. Such reviews have the potential to play an important role in healthcare decision making by providing clinicians and policy makers with reliable and up-to-date information about the accuracy of diagnostic tests. Their impact, however, may be limited if healthcare professionals find the review reports - the main way of communicating the results - inaccessible. By employing the think-aloud method and using the first three DTARs published in the Cochrane library as interview material, we explored how clinicians and policy makers with different levels of background knowledge made sense of such reports.

The results from the study demonstrate that readers unfamiliar with this type of review may experience a range of difficulties which, in some cases, may lead to complete misunderstanding of the results. Some of the identified difficulties - such as those related to poor understanding of test accuracy measures - have been well-documented in previous studies [8,11,12,17,18,20,22,26]. Others relate to the architecture of test accuracy research and the methods specific to DTARs and, as far as we know, have not been previously reported. Although the lack of background knowledge was the main factor in hindering participants’ understanding, the reading strategies they adopted and the way in which information was presented in the reports significantly contributed to the experienced difficulties.

The interviews showed that in their current form the reviews are written on the assumption that they would be read by readers who have good understanding of DTAR methods and do not require special adaptation of the report. Adding ‘accessibility’ features, such as definitions of key terminology, detailed legends to diagrams and text boxes offering accessible interpretations, is left to the discretion of the review authors and, as a result, the accessibility of the three reviews used in the studies varied significantly. It is very likely, however, that the majority of healthcare professionals to whom the results from such reviews might be relevant would not be familiar with DTARs and would find them difficult to understand if the reports were not written with such an audience in mind. If the Cochrane collaboration is serious in its intention to make these reports accessible to a wider readership, then this needs to be reflected in the editorial process which, at present, seems to be concerned mainly with the content of the reviews. This applies not only to the accessibility of the reviews but also to their general readability. While reading the reviews, participants identified a number of issues with the layout and presentation of information that made the reports difficult to read and, in some cases, led to confusion and misinterpretations. Since this affected all participants, regardless of their level of knowledge, it makes it even more important that in the process of preparing the reviews for publication, special attention is paid to their readability, which is not the same as complying with the standard Cochrane format.

Participants’ reports suggest that in the real world, many of them, especially clinicians with little interest in the methodology of the reviews, will read only the abstract. Given the fact that such reading strategy often resulted in misinterpretations, it is particularly important to make the abstracts as explicit and accessible as possible, taking into account the potential misinterpretations discussed in the previous section. The following features might be helpful:

• Presenting the results in more accessible formats, such as frequencies, rather than percentages and false positive and false negative rates rather than just sensitivity and specificity;

• Explicitly defining the roles of the different tests in the review, such as ‘index test’ and ‘reference standard’. This would prompt participants uncertain in their understanding of diagnostic accuracy terminology to look up the respective definitions.

• Careful wording of the conclusions so that readers with limited research experience understand what exactly can be concluded from the results.

• Emphasis on the limitations of the results in terms of validity, reliability and applicability.

Since test accuracy is only one element in the larger puzzle of healthcare decision making, it would be naïve to expect that improving the accessibility of the Cochrane DTARs will automatically result in improved diagnostic decisions. A number of clinicians - with different levels of background knowledge - commented that changes to their practice are most likely to happen as a result of policy decisions resulting in new guidelines rather than from them reading a Cochrane review and deciding that changes are needed. On the other hand, policy decisions are often the final stage of a long process that starts with new evidence changing the perceptions of different groups of stakeholders and leading to actions that eventually will result in changes of the current practice. In this respect, the Cochrane DTARs has a special role to play - if they are accessible to a wider audience they are more likely to catalyze the process of change and to contribute to the prompt update of the current practices.

### Limitations of the study

Given the small and highly diverse sample of the study, the results should be treated with caution and should not be used to make generalizations about the level of understanding of different professional groups. Rather, they should be used to warn authors and editors of Cochrane DTARs about some of the potential difficulties that healthcare professionals and policy makers may encounter when reading such a review and to encourage a debate about the best ways of communicating the results from the Cochrane DTARs to different audiences. Nevertheless, the fact that the sample consisted of more motivated and experienced healthcare professionals suggests that the results might represent the best case scenario and that in the general population of healthcare professionals these difficulties might be much more pronounced.

Undoubtedly, participants’ behaviour was influenced by the setup of the interview and, therefore, the way in which participants read the reviews may not be representative of their ‘real life’ behavior. For example some participants from the ‘expert’ end of the spectrum commented that in ‘real life’ they would take much longer time to read the review, especially if they were to report to a policy making committee, and that they would read some of the sections they skipped during the interview. Most of the participants in the interview sample, however, stated that they would not read a systematic review for longer than one hour, which agrees with the results from our online survey, in which over 80% of the sample (n = 103) stated the same and 50% stated that they would not read a systematic review for longer than half an hour (see Additional file 1). We can also speculate that the interview situation may have had the opposite effect on participants, encouraging them to make an extra effort and to be more diligent in their reading. Many of them commented that in ‘real life’ they would read only the abstract, especially if they felt that the results were inconclusive or unreliable.

Most of the analysis was conducted by the first author and some subjective interpretation may also be present. A portion of the interviews, however, were also watched by the second author, and the results were frequently discussed in the team, to make sure that their interpretation was correct. We also presented some of the interim results to the members of the steering group and received feedback on our interpretations.

## Conclusions

Our results clearly demonstrate that participants who have no previous experience with DTARs may find this type of review challenging and may encounter a range of difficulties leading, in some cases, to complete misunderstanding of the results. These difficulties stem from the interaction between participants’ background knowledge, the reading strategies they employ and the way in which information is presented in the reports. In their present format the Cochrane DTARs are written more for specialists than for readers with basic understanding of DTAR concepts and methods. Making the reviews more accessible by adding a glossary, detailed keys to diagrams, plain language summary boxes and other ‘accessibility’ features is likely to help readers who are not specialists in diagnostic research to make better use of the reviews. However, this will require changes to the process of preparing Cochrane DTARs for publication, with consideration being given not only to the contents of the reviews but also to their readability and accessibility. Since many healthcare professionals may not read the whole report, special care should be taken to make the abstract as explicit and accessible as possible so that even readers not familiar with this type of review understand correctly the main results and their implications.

## Abbreviations

CT: Computed tomography; DTA: Diagnostic test accuracy; DTAR: Diagnostic test accuracy review; MRI: Magnetic resonance imaging; NPV: Negative predictive value; PPV: Positive predictive value; QUADAS: Quality of diagnostic accuracy studies; ROC plot: Receiver operating characteristics plot; SROC plot: Summary receiver operating characteristics plot

## Competing interests

The authors declare that they have no competing interests.

## Authors’ contributions

All authors listed have contributed sufficiently to the project to be included as authors, and all those who are qualified to be authors are listed as authors. ZZ, RG and CH contributed equally to the development of the study’s design, the analysis of data and the editing of the final manuscript. ZZ conducted the interviews and wrote the manuscript. All authors read and approved the final manuscript

## Authors’ information

ZZ is a research fellow at the Peninsula Technology Assessment Group at the University of Exeter Medical School and has experience with qualitative research methods. Currently, he is involved in the CARoTT project (http://clahrc-peninsula.nihr.ac.uk/project/50-cardiac-reviews-of-tests-and-training-carott/full.php) which works in collaboration with the Peninsula Heart and Stroke Network to produce a series of systematic reviews of test accuracy studies on diagnostic tests used in cardiovascular diseases.

RG is a senior lecturer in evidence synthesis at the European Centre for Environment and Human Health, University of Exeter Medical School. She is a social science researcher specializing in systematic review and evidence synthesis and is particularly interested in methods of synthesis for qualitative research. Her current research is focused on the use of a broad range of evidence to investigate complex public health issues.

CH is a professor of public health and clinical epidemiology at the University of Exeter Medical School. He was involved in the setting up and running ARIF, a research dissemination unit at Birmingham University to provide support on finding and interpreting evidence to commissioners of health care in the West Midlands health region. Subsequently, he was involved in health technology assessments, particularly to support the work of NICE, and was appointed Director of West Midlands Health Technology Assessment Collaboration. He was also closely connected with the setting up and development of the National Blood Service Systematic Reviews Initiative in Oxford and was an editor of the NHS HTA program's monograph series for several years. His current work is focused on systematic review methods, health technology assessment and evaluation of tests. He has been involved in the development of the Cochrane diagnostic test accuracy review methods, is a member of the Cochrane DTA editorial team and also a member of NICE’s Diagnostic Assessment Committee.

## Supplementary Material

Additional file 1 Results from the online survey. Click here for file
