# Supplementary material for: A qualitative study into the difficulties experienced by healthcare decision makers when reading a Cochrane diagnostic test accuracy review
Source: Syst Rev. 2013 May 16;2:32. doi: 10.1186/2046-4053-2-32 (PMC3663697; doi:10.1186/2046-4053-2-32)
Supplement: Additional file 1 — Results from the online survey. [file 2046-4053-2-32-S1.docx]

# Additional file 1: Results from the online survey

| **Questions** | **Results: All respondents** | | | | **Results: Volunteers** | | | | |
| --- | --- | --- | --- | --- | --- | --- | --- | --- | --- |
| **Survey Respondents: Total:**   - Professional background: | **103** | | | | **46** (subsequently, 3 declined to take part in an interview) | | | | |
| - - Physiotherapists | 43 | | 44.3% | | 12 | 26.1% | | | |
| - - Researchers (HTA) and Policy makers | 16 | | 16.5% | | 16 | 34.5% | | | |
| - - GPs | 15 | | 15.5% | | 5 | 10.9% | | | |
| - - Radiologists | 8 | | 8.2% | | 4 | 8.7% | | | |
| - - Neurologists | 5 | | 5.2% | | 4 | 8.7% | | | |
| - - Lab scientists | 4 | | 4.1% | | 1 | 8.7% | | | |
| - - Haematologists | 2 | | 2.6% | | 2 | 4.3% | | | |
| - - Stroke physicians | 2 | | 2.6% | | 1 | 8.7% | | | |
| - - Oncologists | 2 | | 2.6% | | 1 | 8.7% | | | |
| - - Nuclear medicine physicians | 1 | | 1.0% | | 0 | 0% | | | |
| - - Spinal surgeon | 1 | | 1.0% | | 0 | 0% | | | |
| - - Internal medicine physician | 1 | | 1.0% | | 0 | 0% | | | |
| - Years in practice (clinical and policy making) | Median: 17.5 years  Interquartile range: 10 – 24 | | | | Median: 14  Interquartile range: 6 - 22 | | | | |
| - Clinical and policy making roles   - Clinicians | 87 | | | 89.6% | 10 | | | 21.7% | |
| - - Participants with both clinical and policy making roles | 55 | | | 56.7% | 17 | | | 37.0% | |
|  |  | | | |  | | | | |
| 1. In your professional practice, how often do you come across test accuracy information, e.g. sensitivity and specificity, likelihood ratios, predictive values etc.? |  |  | | |  | | | |  |
| Very rarely | 9.7 % | 10 | | | 4.3 % | | | | 2 |
| Occasionally | 40.8% | 42 | | | 37.0.8% | | | 17 | |
| Frequently | 49.5% | 51 | | | 58.7% | | | 21 | |
| Answered question |  | 103 | | |  | | | 46 | |
| Skipped |  | 0 | | |  | | | 0 | |
| 2. Do you use test accuracy information to (please tick **all** relevant boxes): |  |  | | |  | | |  | |
| To familiarise yourself with a new test | 61.2 % | 63 | | | 60.9 % | | | 28 | |
| To make diagnostic decisions (e.g. to calculate post-test probability of a condition in an individual patient) | 51.5% | 53 | | | 45.7% | | | 21 | |
| To make decisions about the introduction or purchasing of a new test | 35.9% | 37 | | | 30.4% | | | 14 | |
| To draw up guidelines or similar policy activities | 47.6 % | 49 | | | 54.3% | | | 25 | |
| In the context of your professional development | 62.1% | 64 | | | 58.7% | | | 27 | |
| In relation to academic or research activities | 60.2% | 62 | | | 71.7% | | | 33 | |
| Other (please specific) | 13.6 | 14 | | | 17.4% | | | 8 | |
| Answered the question |  | 103 | | |  | | | 46 | |
| Skipped |  | 0 | | |  | | | 0 | |
| 3. Which of the following test accuracy measures/devices have you come across with when making professional decisions? |  |  | | |  | | |  | |
| Sensitivity and specificity | 95.1% | 98 | | | 97.8% | | | 45 | |
| Predictive values | 79.6% | 82 | | | 91.3% | | | 42 | |
| Likelihood ratios | 71.8% | 74 | | | 84.8% | | | 39 | |
| ROC curves | 46.6% | 48 | | | 69.6% | | | 32 | |
| Other (please specify) | 10.7% | 11 | | | 15.2% | | | 7 | |
| Answered question |  | 103 | | |  | | | 46 | |
| Skipped |  | 0 | | |  | | | 0 | |
| 4. What do you find most challenging when using test accuracy information? | **Narrative summary of results** | | | | | | | | |
| Answers revolved around the following problems: | | | | | | | | | |
| - Regardless of their background (clinicians, policy makers, researchers), respondents who in their professional practice rarely came across DTA information, stated that they often struggled to recall the definitions of basic concepts such as sensitivity and specificity, and their implications for practice; they also found challenging the interpretation of ROC plots. - Researchers reported a number of problems such as poor reporting of information in primary studies; lack of clarity regarding the effects of specific biases on the test accuracy results; and lack of information about the impact of test accuracy on treatment choices and patients’ outcomes. - Policy makers discussed the lack of information about the relevance of test accuracy results to specific health care settings and about the relationship between test accuracy and different outcomes; reliability of information; variability of results reported in different studies; lack of clear presentation of relevant information. - Clinicians discussed issues with applicability (in terms of setting, population etc.) and reliability of reported results; the need to have the relevant information at hand when making decisions; and the general uncertainty about applying test accuracy indices in clinical decision making. | | | | | | | | | |
| Answered question |  | 103 | | |  | | | 46 | |
| Skipped |  | 0 | | |  | | | 0 | |
| 5. Have you ever had any training related to test accuracy measures? |  |  | | |  | | |  | |
| Yes | 55.3% | 57 | | | 52.2% | | | 24 | |
| No | 44.7% | 46 | | | 47.8% | | | 22 | |
| Answered question |  | 103 | | |  | | | 46 | |
| Skipped |  | 0 | | |  | | | 0 | |
| 6. Has this training helped you make better use of diagnostic accuracy information? |  |  | | |  | | |  | |
| Answered question |  | 57 | | |  | | | 24 | |
| Skipped |  | 46 | | |  | | | 22 | |
| 7. Have you ever been involved in a research project or academic activity related to test accuracy? |  |  | | |  | | |  | |
| Yes | 35.0% | 36 | | | 50.0% | | | 23 | |
| No | 65.0% | 67 | | | 50.0% | | | 23 | |
| If ‘Yes’ please provide brief description |  |  | | |  | | |  | |
| Answered question |  | 103 | | |  | | | 46 | |
| Skipped |  | 0 | | |  | | | 0 | |
| 8. Within the last year or so, approximately how many systematic reviews, if any, have you read? |  |  | | |  | | |  | |
| None | 17.2% | 17 | | | 17.4% | | | 8 | |
| One | 2.0% | 2 | | | 2.2% | | | 1 | |
| Two | 9.1% | 9 | | | 15.2% | | | 7 | |
| Three | 13.1% | 13 | | | 8.7% | | | 4 | |
| Four | 4.0% | 4 | | | 4.3% | | | 2 | |
| Five | 5.1% | 5 | | | 0.0% | | | 0 | |
| More than 5 | 48.5% | 48 | | | 52.2% | | | 24 | |
| Answered question |  | 99 | | |  | | | 46 | |
| Skipped |  | 4 | | |  | | | 0 | |
| 9. On average, how much time do you spend on reading a systematic review, which is directly relevant to your professional practice? |  |  | | |  | | |  | |
| N/A | 7.1% | 7 | | | 6.5% | | | 3 | |
| 15 min | 24.2% | 24 | | | 19.6% | | | 9 | |
| 30 min | 27.3% | 27 | | | 21.7% | | | 10 | |
| 45 min | 14.1% | 14 | | | 15.2% | | | 7 | |
| 1 hour | 7.1% | 7 | | | 6.5% | | | 3 | |
| 1-2 hours | 8.1% | 8 | | | 10.9% | | | 5 | |
| More than 2 hours | 12.1% | 12 | | | 19.6% | | | 9 | |
| Answered question |  | 99 | | |  | | | 46 | |
| Skipped |  | 4 | | |  | | | 0 | |
| 10. Which parts of a review which is directly relevant to your practice, do you usually read? |  |  | | |  | | |  | |
| N/A | 4.0% | 4 | | | 4.3% | | | 2 | |
| Abstract | 84.8% | 84 | | | 84.8% | | | 39 | |
| Methods | 63.6% | 63 | | | 73.9% | | | 34 | |
| Results | 68.7% | 68 | | | 73.9% | | | 34 | |
| Discussion | 74.7% | 74 | | | 76.1% | | | 35 | |
| Conclusions | 81.8% | 81 | | | 76.1% | | | 35 | |
| Others | 12.1% | 12 | | | 21.7% | | | 10 | |
| Answered question |  | 99 | | |  | | | 46 | |
| Skipped |  | 4 | | |  | | | 0 | |
| 11. How familiar are you with the methodology of systematic reviews? |  |  | | |  | | |  | |
| Not very familiar (1 on a scale of 1 to 5) | 5.1% | 5 | | | 4.3% | | | 2 | |
| 2 | 14.1% | 14 | | | 6.5% | | 3 | | |
| 3 | 29.3% | 29 | | | 28.3% | | 13 | | |
| 4 | 23.2% | 23 | | | 28.3% | | 13 | | |
| Very familiar (5 on a scale of 1 to 5) | 28.3% | 28 | | | 32.6% | | 15 | | |
| Answered question |  | 99 | | |  | | 46 | | |
| Skipped |  | 4 | | |  | | 0 | | |
| 12. Have you read any of the three Cochrane Diagnostic Tests Accuracy Reviews referred to above? |  |  | | |  | |  | | |
| Yes | 25.3% | 25 | | | 19.6% | | 9 | | |
| No | 74.7% | 74 | | | 80.4% | | 37 | | |
| Leeflang et al 2008 |  | 2 | | |  | | 0 | | |
| Brazzelli et al 2009 |  | 8 | | |  | | 4 | | |
| van der Windt et al 2008 |  | 19 | | |  | | 5 | | |
| Answered question |  | 99 | | |  | | 46 | | |
| Skipped |  | 4 | | |  | | 0 | | |
| 13. How understandable did you find the reviews |  |  | | |  | |  | | |
| - Difficult to understand |  | 0 | | |  | | 0 | | |
| - Generally understandable but some difficulties encountered: |  |  | | |  | |  | | |
| Leeflang et al 2008 |  | 1 out of 2 | | |  | | 0 | | |
| Brazzelli et al 2009 |  | 4 out of 8 | | |  | | 3 out of 4 | | |
| van der Windt et al 2009 |  | 12 out of 19 | | |  | | 4 out of 6 | | |
| - Easy to understand: |  |  | | |  | |  | | |
| Leeflang et al 2008 |  | 1 out of 2 | | |  | | 0 | | |
| Brazzelli et al 2009 |  | 4 out of 8 | | |  | | 1 out of 4 | | |
| van der Windt et al 2009 |  | 7 out of 19 | | |  | | 2 out of 6 | | |
| 14. What difficulties, if any, did you encounter? (more than one answer possible) |  |  | | |  | |  | | |
| No difficulties encountered |  | 12 | | |  | | 3 | | |
| Not familiar with terminology |  | 6 | | |  | | 2 | | |
| Not familiar with structure |  | 3 | | |  | | 2 | | |
| Not familiar with graphical presentation |  | 4 | | |  | | 2 | | |
| Structure not user-friendly |  | 1 | | |  | | 0 | | |
| Insufficient explanations provided |  | 2 | | |  | | 1 | | |
| Conclusions not clear |  | 1 | | |  | | 1 | | |
| Implications for practice not clear |  | 5 | | |  | | | 2 | |
| Please explain and provide additional categories if necessary |  | 8 | | |  | | | 4 | |
| Answered question |  | 24 | | |  | | | 10 | |
| Skipped |  | 79 | | |  | | | 36 | |
| 15. Have you read any other systematic reviews of diagnostic test accuracy studies? |  |  | | |  | | |  | |
| Yes | 34.34% | 34 | | | 37.0% | | | 17 | |
| No | 63.64% | 63 | | | 63.0% | | | 29 | |
| Answered question |  | 99 | | |  | | | 46 | |
| Skipped |  | 4 | | |  | | | 0 | |
| 16. What difficulties, if any, did you encounter? (more than one answer possible) |  |  | | |  | | |  | |
| No difficulties encountered |  | 15 | | |  | | | 8 | |
| Not familiar with terminology |  | 6 | | |  | | | 3 | |
| Not familiar with structure |  | 3 | | |  | | | 1 | |
| Not familiar with graphical presentation |  | 6 | | |  | | | 4 | |
| Structure not user-friendly |  | 2 | | |  | | | 1 | |
| Insufficient explanations provided |  | 5 | | |  | | | 2 | |
| Conclusions not clear |  | 0 | | |  | | | 0 | |
| Implications for practice not clear |  | 10 | | |  | | | 7 | |
| Please explain and provide additional categories if necessary |  | 9 | | |  | | | 6 | |
| Answered question |  | 31 | | |  | | | 46 | |
| Skipped |  | 72 | | |  | | | 0 | |
| 17. In your opinion, how could the accessibility and comprehension of the diagnostic test accuracy reviews be further improved? | **As reported in the survey** | | | | | | | | |
| Ensure consistency of reporting, have very clear guidance for authors. Adequate and better training on methods used, and interpretation.  I don't think I'm qualified to answer this. I sometimes struggle with the statistical methodology and visual presentation of results - but I think this is probably my shortcomings rather than those of the review.  Probably for me to have a refresher course!  I think improvement in the conduct and reporting of the primary studies is needed, and the use of reporting guidelines both for primary and secondary assessments.  I do not recall having problems comprehending diagnostic test accuracy reviews, however I am very familiar with systematic review methods and somewhat familiar with test accuracy research.  I think some clear interpretation of the findings and graphical presentations would be helpful, e.g. rather than reporting the values of sensitivity and specificity, also give some interpretation of the findings and what they mean.  More studies into composite testing and subsequent accuracy and then specific clinical guidelines for best clinical practice for use in organisation/teaching as best available evidence.  Reporting in simpler terms as much as possible and by developing an approach of reporting the impact of each bias that will effect the direction of each test accuracy measures rather than just summarising the quality of studies.  More critical analysis of their own methodology.  More explanatory analysis on statistics performed and more focus on clinical implications of findings and on constructive suggestions (rather than i.e. providing the usual '... moderately or not sufficient evidence on ....).  Unsure! Perhaps by explaining in layman's terms the diagnostic terms that are used in the review, and explaining the results clearly.  An analogy: 'thank you for telling me what the machine does, but tell me more on how to use it'. I think better access to sites on the internet that specialise in reviews on tests. These reviews get published in journals all over the world and as a clinician I do not necessarily have access to all. I don't think not all clinicians, myself included, have the academic understanding to analyse the reviews which then have implications on applying the tests and my willingness to apply the tests.  Perhaps a summary box in the paper explaining the statistical method used and what you are looking for to conclude a useful test or not. Sometimes this is included in the discussion section but not always.  Recognised similar format and method, brief explanation of diagnostic accuracy terms used for those who don't read such papers regularly? Brief explanation of graphs, forest plots, ROC curves etc.  Simple language. | | | | | | | | | |
